# 10 Reasons to be Tantalized by the B73 Maize Genome

**DOI:** 10.1371/journal.pgen.1000723

**Published:** 2009-11-20

**Authors:** Virginia Walbot

**Affiliations:** Stanford University, Stanford, California, United States of America

Why should you read about the maize genome? Now that so many eukaryotic genomes are available, it's easy to be blasé… just another few billion bases, grist for constructing gene trees. Why is this new information, so recently shared, worth considering? I am convinced, as I propose you will be too as you read on, that both geneticists and genome consumers will benefit from the first description of the B73 maize genome [1] and equally so from the companion papers compiled in this special collection (http://collections.plos.org/plosgenetics/maize.php).

The genomic cartographers of maize used a minimal tiling path of 16,000 bacterial artificial chromosomes (BACs) [2] to sequence genes with high precision (often defined by full-length complementary DNAs [cDNAs]; see Soderlund et al. [3]), partially finish repetitive elements, and order both within each BAC. Complementing this and detailed genetic maps accrued over the past century, Zhou et al. [4] developed a tour de force single-molecule optical map by physically anchoring greater than 91,000 restriction sites in the genome.

From historical discoveries to the latest in association mapping of key agronomic traits [5], maize continues to be an important genetic model organism. Now, the authors in this collection have used the freshly minted maize genome to begin to probe some of the most intriguing questions in both genetics and plant biology. With this new genome-wide perspective, we invite you to ponder a sampling of the unsolved questions ripe for investigation.

## 1) Maize Lines Are More Diverse Than the Human–to–Chimp Comparison

Corn geneticists exploit the astonishing allelic diversity of maize for mapping and association tests. In 10,000 years of domestication from the wild relative teosinte, maize has retained and generated allelic diversity and new genes, resulting in greater divergence than is found between two hominids separated by 3.5 million years. Springer et al. [6] compared B73 to another modern inbred and report an unprecedented level of structural diversity—differences in gene copy number and hundreds of genes present in only one line. Soderlund et al. [3] also point out that maize has many genes not found in other higher plants. What selective forces in both the progenitor wild species and modern cultivar have elevated gene generation and allelic diversification (from length polymorphism, single nucleotide changes, and transposon insertion/excision events) orders of magnitude higher in maize than most other plants and animals?

## 2) Inbreeding Depression—Is It Homozygosity Only That Is So Deleterious?

Sequential self-pollination of corn over multiple generations results in progressively smaller plants [7]. Does this simply reflect fixation to homozygosity of some deleterious alleles in each derivative line or epigenetic phenomena? Why are diploidized haploids derived from haploid cells—and hence entirely homozygous—even viable?

## 3) Hybrid Vigor—Reversing Inbreeding Depression: Could It Be Much More Than Restoration of Heterozygosity?

After deriving spindly, short plants through selfing, cross breeding with other inbred lines resulted in tremendous vigor and early flowering. This vigor is the foundation of corn seed production. Hybrid vigor is more than a return to heterozygosity at many loci—is it mostly an epigenetic phenomenon, and, if so, what mediates the resetting of the governing epigenetic marks?

## 4) Centromeres—What's Actually There and What Really Matters?

What are the minimum requirements to be a functional centromere and how do the DNA elements within centromeres evolve rapidly, while maintaining functions over time? Learn details about two maize centromeres in Wolfgruber et al. [8]. The authors set a landmark by traversing the largest centromeres for which functions have been assigned to specific sequence types.

## 5) Recombination and DNA Transposons Target the Same Sequences, but Not Always Genes

In 1931 Creighton and McClintock established that meiotic recombination involves the physical exchange of chromosome segments [9]. This achievement was followed by McClintock's even better known discovery of maize transposable elements in the 1940s. With the genome in hand, Liu et al. [10] demonstrate that both insertion and recombination are associated with epigenetic hallmarks of open chromatin. In particular they show that transposon insertions favor the 5′ ends of genes and that both transposon insertions and recombination frequencies increase as a function of distance from the centromere. Yet not all recombination and insertion occurs in genes. Is “genome action” really centered on genes, and, if not, what makes a non-coding region a hotspot?

## 6) So Many Transposons; So Much Genome Fluidity

The maize genome is more than 80% Class I (reverse transcription required for insertion) retrotransposons with the more famous Class II (DNA is the transposition intermediate) elements such as *Ac/Ds*, *Spm* (discovered by McClintock) and *Mu* elements, which have been so useful for gene tagging, comprising a small fraction of the genome. Extending studies on small regions of the maize genome, new insights are reported on the diversity and location preferences for the hundreds of families of retroelements (most newly described by Baucom et al. [11]). Born in bursts, identical retroelements disperse in the genome and provide an archeological tool for dating events, such as subsequent insertions of younger elements. What causes these periodic assaults by armies of retroelements? Wei et al. [12] analyzed one 22-Mb region of Chromosome 4, taking care to track gene fragments captured by TE and delivered to this chromosome from other genomic locations. Are transposons rearranging gene fragments the primary mechanism for generating new genes in maize?

## 7) Paramutation—a Classic Violation of Mendel's Laws Can Now Be Explored Genome-Wide

This enigmatic phenomenon, documented by Brink in the 1950s (reviewed by Chandler and Stam [13]), describes the ability of some alleles at transcription factor loci to permanently down-regulate the expression of other alleles. The phenomenon is mediated epigenetically and does not require synteny in maize. Why do some alleles evolve with the ability to turn off expression? Is paramutation widespread, with “killer” alleles present for many loci? Recently recognized in humans [14] and mice [15],[16], how general is paramutation in eukaryotes?

## 8) Imprinting

Discovered 40 years ago, this parent-of-origin influence on expressivity is one maize observation that was quickly recognized and served as a sufficient explanation for many puzzling cases in mammals [17]. In plants, imprinting is exhibited not in the embryo but in the accessory seed tissue (the endosperm) formed during double fertilization. Early success of the endosperm requires an appropriate chromosomal constitution—typically two maternal and one paternal genome—with imprinting modulating the effective dosage of particular alleles of genes. Incorrect imprinting of the endosperm failure dooms the embryo, but why use imprinting as a temporary means of controlling gene expression rather than selection for transcription factor or promoter combinations for appropriate gene expression?

## 9) Agents of RNA-Based Regulation—Are They Controlling All of the Epigenetic Phenomena?

The importance of small RNAs as regulatory agents can hardly be overstated, and, based on deep, short-read sequencing reported by Zhang et al., these elements abound in the maize genome [18]. Specific cases had already been discovered and cloned by geneticists as key regulatory loci in developmental pathways, but now the beginning of the “big picture” is available. Similarly, the impact of a loss-of-function mutant in the RNA-directed DNA methylation silencing pathway was interrogated. *mop1* was first identified as required in paramutation and as a suppressor of *Mu* transposons, but in its absence thousands of genes are affected and, surprisingly, most are down-regulated [19]. Did control of transposons evolve into cellular mechanisms for fine-tuning gene expression patterns?

## 10) Most Importantly, Corn Is a Key Element in the Global Food Economy

American farmers grew 1 metric ton of corn per citizen in 2008; worldwide corn feeds directly (starch, high fructose corn syrup, oil) or indirectly (through meat) about 1 billion people. Thousands of products (ethanol, coatings for paper and cloth, biodegradable plastics, corn cob pipes, etc.) derive from this renewable, typically locally available staple. The overarching question now is how we can use the unprecedented genetic tool that the maize genome offers to improve corn productivity per unit of land while reducing inputs such as water and fertilizer so that we can sustain humanity's food requirements, while also decreasing the negative impacts of agriculture on the Earth.

The genetic puzzles now yielding solutions, from genetic and now genome-wide analysis, are intriguing. If you like to compartmentalize and focus only on your own species, I'll remind you again that phenomena such as transposons, paramutation, and imprinting were all discovered in maize and ascribed by many as “corn-specific” cases until someone (this could be you) recognized parallel phenomena in animals or fungi. Thus, I challenge you to think deeply as you read about hybrid vigor, new insights into transposon types and distribution, the abundance of very short FLcDNAs encoding predicted peptides, and the many other “genetic jewels” contained in this collection.
